# The landscape of spiritual health and spirituality in Canada: A scoping review protocol

**DOI:** 10.1371/journal.pone.0309294

**Published:** 2024-08-29

**Authors:** Helana Marie Boutros, Merna Mina, Nelly Van Doorn-Harder, Maurita T. Harris

**Affiliations:** 1 Department of Health, Society and Aging, McMaster University, Hamilton, Ontario, Canada; 2 ICES, Toronto, Ontario, Canada; 3 Faculty of Liberal Arts, Wilfrid Laurier University, Waterloo, Ontario, Canada; 4 Department of Health Sciences, Wilfrid Laurier University, Waterloo, Ontario, Canada; 5 Department for the Study of Religions, Wake Forest University, Winston-Salem, North Carolina, United States of America; University of Alabama at Birmingham, UNITED STATES OF AMERICA

## Abstract

**Introduction:**

Currently, literature on spiritual health remains limited. Even more so, literature on spiritual health remains limited in Canada. This is exacerbated by the fact that spiritual health is a term that remains widely contested with no concrete definition. Meanwhile, the semantic connection between “religion” and “spirituality” remains ambiguous in the West and scholars hold different positions. In this paper, we outline our scoping review protocol to describe the current landscape of spiritual health and spirituality (in relation to health) research in Canada, including the meaning and understanding behind these two concepts.

**Methods and analysis:**

This protocol for our scoping review is documented in accordance with the PRISMA-P reporting guidelines and adheres to Arksey and O’Malley’s scoping review methodology. We will conduct a search strategy across select electronic databases and review the reference lists of chosen papers. Two reviewers, HMB and MM, will independently and blindly screen all title/abstracts and full-text studies for eligibility. Any Canadian-situated studies that centrally mention and focus on “spirituality” or “spiritual health” will be included. Relevant variables will be extracted through an iterative process, with the data charting being continuously reviewed and refined. Findings from this scoping review will support the future of health research and conceptual expansion of health beyond the mental, physical and social. Approval from a research ethics board is not required, nor has it been obtained, as the data is derived from journal articles and academic publications.

## Background

Spiritual health is conceptualized, theorized, and studied substantially against two disciplines in the literature: health promotion and nursing practice. This is not to say that theology and philosophy do not play a role in contributing to the current understanding of spiritual health. There is an inevitable distinctive flavour of these disciplines in nursing practice and health promotion, but it is a flavour more so subconsciously underpinned instead of explicitly expressed [1–3].

Today, the term “spiritual health” is a growing linguistic term that is being used across different countries and different continents, having had linguistic use since the 1980s [4–17]. Furthermore, there have been multiple studies whose research objectives define spiritual health [6,11,12,14], only one of which is based in Canada [18]. However, despite this growing emphasis on wholeness and holism, there remains a noticeable absence of discussion surrounding spirituality and spiritual health [19].

It can be argued that the re-introduction in the 1980s of spiritual health in disciplines, such as health promotion and nursing practice, is precipitated by a convicting desire to counter Descartes-infused biomedical practices within healthcare and health promotion practice. In the 17^th^ century, Descartes argued that the human body is mechanistically understood and conceptualized, independent of the mind [14,20,21]. This philosophy led to mind-body dualism and its mutational offshoots contributed to 21st-century biomedicine today, where biomedicine particularly focuses on relieving physical symptoms of disease and illness instead of focusing on etiology and prevention [14,20].

While the negative implications of the current biomedical approach arguably placed a new enthusiasm for spiritual health on the map, health was not always biomedically conceptualized [22]. Historically, a holistic understanding of health was spawned in Ancient Greek medicine [20]. Unlike its modern biomedical counterpart, Ancient Greek medicine argued that it was insufficient to define health as the absence of disease and unpleasant symptoms [20]. Instead, Ancient Greeks asserted that health is more wholly characterized by an individual’s sound spiritual base and a satisfying, full life [11,20,22]. Therefore, the physician’s role in Ancient Greek medicine was centralized around restoring order instead of simply removing unpleasant symptoms [20]. For example, Hippocrates asserted that a person’s worldview or way of life is a normative feature for one’s health. This implies that a person’s beliefs and value systems are decisive factors for their health. Therefore, it is reasonable to assume that in-depth awareness of the multi-dimensional role that beliefs and value systems play leads to the integration of these beliefs in one’s daily life, which impacts one’s well-being [11,23]. It is also reasonable to assume that if a person’s beliefs and value systems are dichotomous and logically inconsistent with their life patterns and behaviour, wholeness cannot be achieved, and their well-being may be negatively impacted [11].

As part of a slow-growing paradigm shift, the World Health Organization (WHO) expanded its definition of health “from the absence of disease and illness” to include an element of wholeness where health is defined as “a state of complete physical, mental and social well-being” [15]. However, WHO has yet to include a spiritual dimension of health [15,19]. In this sense, the dimension of spiritual health today remains Hippocratic in an expansionist way but not necessarily a normative feature in health, even if there is a competing shift away from the biomedical approach to health. While biomedical approaches remain strong, there is a growing re-permeation of “wholeness” in health circles: it is the helping disciplines of nursing practice and health promotion that illuminate the resurgence and need for spiritual health today [7,13,14,24].

Literature on spiritual health remains limited across many countries, including Canada. The most recent reviews on spiritual health were published in 2007 and 2022 and identified several gaps in spiritual health research [1,9]. This is exacerbated by the fact that spiritual health is a term that remains widely contested with no concrete definition. Meanwhile, the semantic connection between “religion” and “spirituality” remains ambiguous in the West and scholars hold different positions [24–26]. Critical to the wellness and health discussion is the discourse on “spiritual, but not religious” or “spiritual and religious.” The definition of spirituality arguably has become more elastic especially now since people no longer wish to conform to established religious structures such as churches, mosques and the like. This has important implications since Canada is considered a multicultural country whose citizens practice a variety of different religions and spiritualties and uphold diverse worldviews. Thus, a knowledge synthesis of spiritual health and spirituality (in relation to health) is invaluable.

This scoping review protocol aims to build on the previous knowledge synthesis work of Litalien et al., 2022, who conducted a systematic literature review exploring the influence of religiosity and spirituality on health across Canada [27]. While Litalien et al. focus on the influence of religiosity and spirituality on health, our scoping review specifically focuses on the relationship between spirituality and health (its influence, its conceptualization and so forth), and the implicit relationship between spirituality and religion in the context of Canada today. Furthermore, we build on Litalien et al.’s search strategy by expanding it to enhance its robustness. This scoping review protocol also aligns with previous calls to action–in health promotion and curricula–to emphasize and promote spiritual health in the Canadian context [18,19,28]. Since spiritual health is a growing term, it is intellectually reasonable to assume that research on spiritual health may not necessarily be linguistically framed in those exact words. Instead, many researchers may do work related to spiritual health, but may reciprocally relate spirituality to health [24,25,29,30]. Thus, this scoping review is exploratory beyond the term, “spiritual health” and explores spirituality in relation to other dimensions of health as well. Finally, this scoping review protocol heeds the call that spirituality is salient to health [19,24].

## Scoping review objectives

The main aim of this scoping review will be to outline and describe the current landscape of spiritual health and spirituality (in relation to health) research in Canada, including the meaning and understanding behind these two concepts. Secondary aims will include determining the proportion of studies that demonstrate a link between (a) spirituality and health to religion and (b) spiritual health to religion. Based on these objectives, this scoping review will be exploratory.

### Methods and analysis

A scoping review will be conducted to examine the current landscape of spirituality and spiritual health and wellness research [31]. This approach was selected because it enables a broader examination of spirituality and spiritual health, rather than concentrating on specific outcomes [32]. As a form of knowledge synthesis, conducting a scoping review will capture developments in spiritual health and spirituality research across disciplines. All in all, our aim is to gather recent research on spirituality and spiritual health, which can guide future projects in this field [33].

This scoping review is an initial preliminary step to understanding spiritual health in Canada. The results from this scoping review will inform the study design of future research projects and future work on spirituality and spiritual health across Canada. Research ethics board approval is not required since data are from publications or publicly shared reports.

### Protocol design

This scoping review will adhere to Arksey and O’Malley’s methodology for scoping review [31]. Based on this methodology, there are six different stages in developing a scoping review: (1) identifying the research question; (2) identifying relevant studies; (3) selecting studies; (4) charting the data; (5) collating, summarizing, and reporting the results and (6) consulting with relevant stakeholders (which is optional according to Arksey and O’Malley). Thus, we will adhere to Arksey and O’Malley’s methodology while incorporating Levac et al.’s approach, namely, to consult with stakeholders as a knowledge translation requirement [34]. In our case, we will consult Dr. Nelly Van Doorn-Harder throughout the scoping review process. Nelly van Doorn-Harder is a scholar of religion. Presently, she is a professor at Wake Forest University (North Carolina, USA) and the Vrije Universiteit (Amsterdam, Netherlands). Her expertise in religious studies, interfaith engagement and anthropology background will inform this work and ensure that this scoping review is relevant for multiple communities [35]. To present the search findings, the Preferred Reporting Items for Systematic Reviews and Meta-Analysis will be utilized [36].

### Stage 1: Identifying the research question

The research questions are:

RQ1: What research currently exists on spirituality (in relation to health) and spiritual health in Canada?
RQ2: How are spirituality (in relation to health) and spiritual health conceptualized and understood?
RQ3: Does the mention of spirituality converge with the mention of religion in the context of health?

### Stage 2: Identifying relevant studies—*Search strategy and information sources*

This stage will be characterized by an iterative process. This will comprise of reviewing the literature, refining the search strategy, and incorporating additional sources found in the reference lists of selected papers. Initial screening of titles and abstracts will precede the full-text review of chosen studies. The research team has collaboratively developed and finalized the search strategy, as well as the eligibility criteria. The study is scheduled to take place from April 2024 to August 2024.

Relevant studies for this review will be identified through a comprehensive search conducted in the following electronic databases: ProQuest Databases (including MEDLINE and APA PsychINFO), EBSCO Host Databases, Scopus, Embase, Web of Science, Anthropology Plus, and PubMed. The search strategy will involve systematically combining keywords and Boolean operators (AND/OR) across each database.

**Search String 1:** (spirituality or spiritual needs or spiritual care or spiritu*)

**Search String 2:** (Canada or Canadian or Canadians or in Canada)

**Search String 3:** (health or wellbeing or well being or well-being or quality of life or wellness or physical health or social health or spiritual health or intellectual health or emotional health or financial health or environmental health or occupational health or intellectual health)

Particularly for Search String 3, we ensured that it encapsulates at least eight dimensions of well-being, as per Colorado State University’s “Health Education and Prevention” information [37]. The full search strategy across all databases is provided in this protocol’s supplementary material (see S1 Table). Search results will be saved as.ris files and imported into Covidence for study screening and data management [38].

To ensure a comprehensive literature coverage of spirituality and spiritual health in Canada is attained, a Google search will also be conducted (for the first 100 hits) to identify relevant journal articles and academic publications in line with this scoping review. To identify additional studies that are in line with this scoping review’s inclusion and exclusion criteria, forward and backward citation searches of all included publications will be conducted.

### Stage 3: Study selection

The review process will involve two stages of screening: first, a review of titles and abstracts, and second, a full-text review. Two researchers (HMB and MM) will independently screen all imported citations for titles and abstracts in a double-blind manner, applying the predefined inclusion and exclusion criteria. Covidence will handle duplicate removal prior to title and abstract screening, and any remaining duplicates will be manually removed by HMB and MM. In cases of disagreement/ conflict on title and abstract inclusion, the third reviewer (MTH) will review the studies and make a final decision to exclude ineligible studies and resolve conflicts on Covidence. Studies included by both HMB and MM will proceed to full-text review. During the full-text review stage, the same two reviewers (HMB and MM) will independently screen studies against the same predefined inclusion and exclusion criteria. Any discrepancies in full-text inclusion will be reviewed by the third reviewer (MTH), who will make final decisions to exclude ineligible studies and resolve conflicts on Covidence, prior to proceeding to the data extraction phase.

### Inclusion criteria

Published in the English language

Peer-reviewed journal articles

Research is focused within the Canadian context

Available electronically in full text

Current studies that mention and focus on any type of “spirituality” or “spiritual health”

Must mention some aspect of health, well-being or wellness (it could also mention an aspect antithetical to health, such as illness, distress, etc.)

All articles until the present day

### Exclusion criteria

Grey literature

Printed in languages other than English

Papers without evaluation and reporting of results/ or knowledge contributions

Exclude any studies loosely tied to spirituality/health and spiritual health. For example, a study focusing on the correlates of smoking, mentioning briefly that “spiritual and social health are important.”

### Stage 4: Data collection

Data extraction for study details will be organized in a charting table on an Excel spreadsheet for full-text studies, relevant forward and backward citations of full-text studies and hand searched studies. The team will employ an iterative approach through team meetings and email exchanges to review, refine and consistently update the data extraction table. Relevant variables will be extracted, including, but not limited to author(s), year of publication, journal of publication, open/ closed access, year of data collected (if applicable), methodology, findings, and knowledge contributions.

### Stage 5: Data summary and synthesis of results

The search results will be reported using the Preferred Reporting Items for Systematic reviews and Meta-Analysis extension Protocols (PRISMA-P) as per PLOS ONE knowledge synthesis journal requirements (See S2 Table) [39]. As a scoping review aims to outline key concepts and define the boundaries of a topic or field, this review will not conduct critical appraisal of the included studies [31,32]. Instead, this scoping review will offer a comprehensive overview of all studies that are included [31,32]. Data will be synthesized and summarized from our data extraction table to create a narrative report detailing available data on the following themes: (1) the extent to the relationship between spirituality and religion in Canada is intertwined/ interchangeable or disparate (i.e. may present themselves in micro discourse analysis of research and personal narratives regarding the relationship between spirituality and religion), (2) whether religion and/or spirituality contains negative connotations (i.e. public discourse, literature, historical contexts where religion or spirituality reflected social conflict, oppression or discrimination) (3) theoretical versus empirical paper, (4) the extent that spirituality been conceptualized in relation to health (i.e. theoretical models, empirical research, interventions or therapeutic approaches that integrate spirituality into healthcare settings) and (5) the context in which religion is mentioned (i.e. immigration, colonialism, religious rituals or practices observed in public spaces etc.), (6) cultural influences (i.e. indigenous perspectives, immigrant communities, geographical differences etc.) and (7) interfaith dialogue (between different religious and spiritual groups in Canada).

### Stage 6: Consultation

This scoping review will be fundamentally informed and supported by faculty members and academics within religious studies, health anthropology and/or interdisciplinary health academic backgrounds. At Stage 3 of the research, researchers who actively research within these particular fields will be contacted to gain a deeper understanding of spirituality and spiritual health within the context of Canada. This will be based on authorship frequency relative to other authors in the research and Religious Studies and Health Promotion stakeholders within HMB’s network. These two critical pathways to consultation will lead to the co-interpretation and enrichment of extracted knowledge alongside HMB and MM.

## Supporting information

S1 Table Detailed search strategy across all selected databases for this scoping review protocol. Detailed search strategy across all selected databases for this scoping review protocol.
(PDF)

S2 Table Completed PRISMA-P checklist for scoping review protocol. Completed PRISMA-P checklist for scoping review protocol.
(PDF)
