# Correction: The landscape of spiritual health and spirituality in Canada: A scoping review protocol

**DOI:** 10.1371/journal.pone.0341783

**Published:** 2026-01-27

**Authors:** Helana Marie Boutros, Merna Mina, Nelly Van Doorn-Harder, Maurita T. Harris

In the Background section, there is an error in the second sentence of the third paragraph. The correct sentence is: In the 17th century, Descartes argued that the human body should be mechanistically understood and conceptualized, independent of the mind [14,20,21].

In the Background section, there is an error in the first sentence of the first paragraph. The correct sentence is: As part of a slow-growing paradigm shift, the World Health Organization (WHO), since its founding in 1948, has defined health as not merely“the absence of disease and illness,” but as “a state of complete physical, mental and social well-being” [15].

In the Protocol Design subsection of the Scoping review objectives, there is an error in the first sentence of the first paragraph. The correct sentence is: This scoping review will adhere to Arksey and O’Malley’s methodology for scoping reviews [31].

In the Stage 5: Data summary and synthesis of results subsection of the Scoping review objectives, there is an error in the fourth sentence. The correct sentence is: Data will be synthesized and summarized from our data extraction table to create a narrative report detailing available data on the following themes: (1) the extent to the relationship between spirituality and religion in Canada is intertwined/ interchangeable or disparate (i.e., may present themselves in micro discourse analysis of research and personal narratives regarding the relationship between spirituality and religion), (2) whether religion and/or spirituality contains negative connotations (i.e., public discourse, literature, historical contexts where religion or spirituality reflected social conflict, oppression or discrimination) (3) theoretical versus empirical paper, (4) the extent that spirituality has been conceptualized in relation to health (i.e., theoretical models, empirical research, interventions or therapeutic approaches that integrate spirituality into healthcare settings), (5) the context in which religion is mentioned (i.e., immigration, colonialism, religious rituals or practices observed in public spaces etc.), (6) cultural influences (i.e., indigenous perspectives, immigrant communities, geographical differences etc.) and (7) interfaith dialogue (between different religious and spiritual groups in Canada).

S1 and S2 Tables are incorrect. Please see the correct S1 and S2 Tables here.

## Supporting information

S1 Table Detailed search strategy across all selected databases for this scoping review protocol. (PDF)

S2 Table Completed PRISMA-P checklist for scoping review protocol. (PDF)
