# Supplementary material for: The landscape of spiritual health and spirituality in Canada: A scoping review protocol
Source: PLoS One. 2024 Aug 29;19(8):e0309294. doi: 10.1371/journal.pone.0309294 (PMC11361655; doi:10.1371/journal.pone.0309294)
Supplement: S1 Table — Detailed search strategy across all selected databases for this scoping review protocol. (PDF) [file pone.0309294.s001.pdf]

PLOS

Lorem Ipsum

Lorem ipsum dolor sit amet, consectetur adipiscing elit. Mauris maximus fringilla ligula, in malesuada erat tempor ac. Quisque dapibus posuere turpis, vel aliquam massa vehicula non.

EBSCO Host Databases
((spirituality or spiritual needs or spiritual care or spiritu*) AND (Canada or Canadian or Canadians or in Canada)) AND (health or wellbeing or well being or well-being or quality of life or wellness or physical health or social health or spiritual health or intellectual health or emotional health or financial health or environmental health or occupational health or intellectual health))<br>Limiters - Peer Reviewed; Publication Type: Academic Journal; Publication Type: Academic Journal; Language: English<br><br>Expanders - Apply equivalent subjects<br><br>Search modes - Boolean/Phrase
**Scopus**
((spirituality OR spiritual AND needs OR spiritual AND care OR spiritu*) AND (canada OR canadian OR canadians OR in AND canada) AND (health OR wellbeing OR well AND being OR well-being OR quality AND of AND life OR wellness OR physical AND health OR social AND health OR spiritual AND health OR intellectual AND health OR emotional AND health OR financial AND health OR environmental AND health OR occupational AND health OR intellectual AND health)) AND (LIMIT-TO (DOCTYPE , "ar")) AND (LIMIT-TO (LANGUAGE , "English")) AND (LIMIT-TO (SEARCH WITHIN , "Article title, Abstract, Keywords"))
**EMBASE**
((spirituality or spiritual) and needs) or spiritual) and care) or spiritu*) and (canada or canadian or canadians or in) and canada) and (health or wellbeing or well) and being) or well-being or quality) and of and life) or wellness or physical) and health) or social) and health) or spiritual) and health) or intellectual) and health) or emotional) and health) or financial) and health) or environmental) and health) or occupational) and health) or intellectual) and health)).mp. [mp=title, abstract, heading word, drug trade name, original title, device manufacturer, drug manufacturer, device trade name, keyword heading word, floating subheading word, candidate term word] Limit to English Language
**MEDLINE (through OVID)**
((spirituality or spiritual needs or spiritual care or spiritu*) and (Canada or Canadian or Canadians or in Canada) and (health or wellbeing or well being or well-being or quality of life or wellness or physical health or social health or spiritual health or intellectual health or emotional health or financial health or environmental health or occupational health or intellectual health)).mp. [mp=title, book title, abstract, original title, name of substance word, subject heading word, floating sub-heading word, keyword heading word, organism supplementary concept word, protocol supplementary concept word, rare disease supplementary concept word, unique identifier, synonyms, population supplementary concept word, anatomy supplementary concept word] Limit to English Language
**CINAHL**
AB (spirituality or spiritual needs or spiritual care or spiritu*) AND AB (Canada or Canadian or Canadians or in Canada) AND AB (health or wellbeing or well being or well-being or quality of life or wellness or physical health or social health or spiritual health or intellectual health or emotional health or financial health or environmental health or occupational health or intellectual health)<br>Search modes - Boolean/Phrase
