# Supplementary material for: The landscape of spiritual health and spirituality in Canada: A scoping review protocol
Source: PLoS One. 2024 Aug 29;19(8):e0309294. doi: 10.1371/journal.pone.0309294 (PMC11361655; doi:10.1371/journal.pone.0309294)
Supplement: S2 Table — Completed PRISMA-P checklist for scoping review protocol. (PDF) [file pone.0309294.s002.pdf]

PLOS

Lorem Ipsum

Lorem ipsum dolor sit amet, consectetur adipiscing elit. Mauris maximus fringilla ligula, in malesuada erat tempor ac. Quisque dapibus posuere turpis, vel aliquam massa vehicula non.

PRISMA-P (Preferred Reporting Items for Systematic review and Meta-Analysis Protocols) 2015 checklist: recommended items to address in a systematic review protocol*

Section and topic	Item No	Checklist item	
**ADMINISTRATIVE INFORMATION**			
Title:			
Identification	1a	Identify the report as a protocol of a systematic review	See page 1
Update	1b	If the protocol is for an update of a previous systematic review, identify as such	Protocol is not an update
Registration	2	If registered, provide the name of the registry (such as PROSPERO) and registration number	Protocol is not registered
Authors:			
Contact	3a	Provide name, institutional affiliation, e-mail address of all protocol authors; provide physical mailing address of corresponding author	See page 1
Contributions	3b	Describe contributions of protocol authors and identify the guarantor of the review	See page 8
Amendments	4	If the protocol represents an amendment of a previously completed or published protocol, identify as such and list changes; otherwise, state plan for documenting important protocol amendments	Protocol is not an amendment
Support:			
Sources	5a	Indicate sources of financial or other support for the review	See page 8
Sponsor	5b	Provide name for the review funder and/or sponsor	Not applicable; See page 8
Role of sponsor or funder	5c	Describe roles of funder(s), sponsor(s), and/or institution(s), if any, in developing the protocol	Faculty from different institutions informed the study design; see page 5
**INTRODUCTION**			
Rationale	6	Describe the rationale for the review in the context of what is already known	See pages 3-4
Objectives	7	Provide an explicit statement of the question(s) the review will address with reference to participants, interventions, comparators, and outcomes (PICO)	For research questions, see page 5; for reference to PICO, see eligibility criteria on page 7
**METHODS**			
Eligibility criteria	8	Specify the study characteristics (such as PICO, study design, setting, time frame) and report characteristics (such as years considered, language, publication status) to be used as criteria for eligibility for the review	See page 7
Information sources	9	Describe all intended information sources (such as electronic databases, contact with study authors, trial registers or other grey literature sources) with planned dates of coverage	See page 6
Search strategy	10	Present draft of search strategy to be used for at least one electronic database, including planned limits, such that it could be repeated	Search strategy included on page 6; all search strategies across all electronic databases is included this protocol's supplementary material (S1FIle); this is also referenced on page 6
Study records:			
Data management	11a	Describe the mechanism(s) that will be used to manage records and data throughout the review	

For screening, data will be managed through Covidence - see page 6
For data charting, data will recorded on an Excel spreadsheet document - see page 7

PRISMA-P-checklist_ Spiritual Health scoping review_202408144932-1.tif This is a preview of your figure rendered on a simulated PLOS journal page.

Maecenas ac est sit amet odio sollicitudin euismod. In risus odio, convallis a neque ac, varius ultricies arcu. Vestibulum et quam iaculis, ultricies odio et, molestie magna. Suspendisse vehicula purus id turpis eleifend, et convallis dui dignissim. Praesent tempus elit a metus sollicitudin, sed fringilla nulla porttitor. Nullam in tempus massa. Nunc maximus magna massa, nec volutpat risus rhoncus ut. Fusce quis ante sem. Aenean nulla nibh, tempus sit amet rhoncus at, eleifend vel risus. Sed dictum, sem ultrices elementum pharetra, lacus diam volutpat orci, scelerisque semper dui lacus ut enim.

Suspendisse in nunc id lacus commodo consequat. Proin semper aliquam varius. Fusce vitae neque aliquam nisi ultrices sodales vitae ut enim. Vivamus nec dictum ipsum. Sed condimentum ante eu urna tincidunt tincidunt. In ac lacus nec ipsum viverra volutpat posuere vel lacus. Class aptent taciti sociosqu ad litora torquent per conubia nostra, per inceptos himenaeos. Morbi rhoncus ipsum quis lorem hendrerit, at vulputate massa tempus. Ut arcu nisl, gravida vitae risus ultricies, porta venenatis massa. Cras dignissim, enim at faucibus aliquam, sapien nisl eleifend dolor, vel mollis nulla nisi id ipsum. Pellentesque vehicula ultricies risus sit amet faucibus. Praesent sit amet mi ac est faucibus accumsan. Praesent pulvinar sit amet orci auctor feugiat.

Phasellus vitae congue est. Duis rutrum iaculis nunc, sed sollicitudin neque eleifend nec. Pellentesque ac nisi eget tortor imperdiet sagittis ut in orci. Mauris porta convallis euismod. Donec in ultricies urna, nec interdum lectus. Nullam sit amet finibus augue, eget rutrum metus. Nam faucibus, urna ac finibus eleifend, neque nisi lobortis ante, at pharetra purus purus sed urna. Curabitur sit amet dui at enim porta posuere non vehicula ligula. Suspendisse potenti. Vestibulum arcu magna, vulputate a massa ac, molestie tincidunt dui.

Donec id tempus lacus, sed tristique nulla. Nullam rutrum risus ut pharetra porttitor. Nam mattis dolor erat, sed volutpat est mattis sed. Suspendisse eu porta tellus. Cras gravida velit sed maximus fermentum. Fusce vitae metus commodo, sagittis nunc sed, faucibus nunc. Integer iaculis quam mattis, luctus neque in, viverra magna.

Lorem Ipsum

Lorem ipsum dolor sit amet, consectetur adipiscing elit. Mauris maximus fringilla ligula, in malesuada erat tempor ac. Quisque dapibus posuere turpis, vel aliquam massa vehicula non.

Selection process	11b	State the process that will be used for selecting studies (such as two independent reviewers) through each phase of the review (that is, screening, eligibility and inclusion in meta-analysis) See page 6
Data collection process	11c	Describe planned method of extracting data from reports (such as piloting forms, done independently, in duplicate), any processes for obtaining and confirming data from investigators See page 7
Data items	12	List and define all variables for which data will be sought (such as PICO items, funding sources), any pre-planned data assumptions and simplifications See page 7-8
Outcomes and prioritization	13	List and define all outcomes for which data will be sought, including prioritization of main and additional outcomes, with rationale See page 7-8
Risk of bias in individual studies	14	Describe anticipated methods for assessing risk of bias of individual studies, including whether this will be done at the outcome or study level, or both; state how this information will be used in data synthesis for the purpose of this scoping review, risk of bias will not be assessed
Data synthesis	15a	Describe criteria under which study data will be quantitatively synthesised Not applicable
	15b	If data are appropriate for quantitative synthesis, describe planned summary measures, methods of handling data and methods of combining data from studies, including any planned exploration of consistency (such as I^2, Kendall's τ) Not applicable
	15c	Describe any proposed additional analyses (such as sensitivity or subgroup analyses, meta-regression) Not applicable
	15d	If quantitative synthesis is not appropriate, describe the type of summary planned See page 7
Meta-bias(es)	16	Specify any planned assessment of meta-bias(es) (such as publication bias across studies, selective reporting within studies) Not applicable
Confidence in cumulative evidence	17	Describe how the strength of the body of evidence will be assessed (such as GRADE) Not applicable since this is a scoping review not a systematic review

*** It is strongly recommended that this checklist be read in conjunction with the PRISMA-P Explanation and Elaboration (cite when available) for important clarification on the items. Amendments to a review protocol should be tracked and dated. The copyright for PRISMA-P (including checklist) is held by the PRISMA-P Group and is distributed under a Creative Commons Attribution Licence 4.0.**

From: Shamseer L, Moher D, Clarke M, Ghersi D, Liberati A, Petticrew M, Shekelle P, Stewart L, PRISMA-P Group. Preferred reporting items for systematic review and meta-analysis protocols (PRISMA-P) 2015: elaboration and explanation. BMJ. 2015 Jan 2;349(jan02 1):g7647.

test-test-1 This is a preview of your figure rendered on a simulated PLOS journal page.

Maecenas ac est sit amet odio sollicitudin euismod. In risus odio, convallis a neque ac, varius ultricies arcu. Vestibulum et quam iaculis, ultricies odio et, molestie magna. Suspendisse vehicula purus id turpis eleifend, et convallis dui dignissim. Praesent tempus elit a metus sollicitudin, sed fringilla nulla porttitor. Nullam in tempus massa. Nunc maximus magna massa, nec volutpat risus rhoncus ut. Fusce quis ante sem. Aenean nulla nibh, tempus sit amet rhoncus at, eleifend vel risus. Sed dictum, sem ultrices elementum pharetra, lacus diam volutpat orci, scelerisque semper dui lacus ut enim.

Suspendisse in nunc id lacus commodo consequat. Proin semper aliquam varius. Fusce vitae neque aliquam nisi ultrices sodales vitae ut enim. Vivamus nec dictum ipsum. Sed condimentum ante eu urna tincidunt tincidunt. In ac lacus nec ipsum viverra volutpat posuere vel lacus. Class aptent taciti sociosqu ad litora torquent per conubia nostra, per inceptos himenaeos. Morbi rhoncus ipsum quis lorem hendrerit, at vulputate massa tempus. Ut arcu nisl, gravida vitae risus ultricies, porta venenatis massa. Cras dignissim, enim at faucibus aliquam, sapien nisl eleifend dolor, vel mollis nulla nisi id ipsum. Pellentesque vehicula ultricies risus sit amet faucibus. Praesent sit amet mi ac est faucibus accumsan. Praesent pulvinar sit amet orci auctor feugiat.
